# The Role of NOD Mice in Type 1 Diabetes Research: Lessons from the Past and Recommendations for the Future

**DOI:** 10.3389/fendo.2018.00051

**Published:** 2018-02-23

**Authors:** Yi-Guang Chen, Clayton E. Mathews, John P. Driver

**Affiliations:** ^1^Department of Pediatrics, Medical College of Wisconsin, Milwaukee, WI, United States; ^2^Department of Pathology, Immunology and Laboratory Medicine, College of Medicine, University of Florida, Gainesville, FL, United States; ^3^Department of Animal Sciences, University of Florida, Gainesville, FL, United States

**Keywords:** NOD mouse, type 1 diabetes, preclinical, congenic, genetics, gene editing

## Abstract

For more than 35 years, the NOD mouse has been the primary animal model for studying autoimmune diabetes. During this time, striking similarities to the human disease have been uncovered. In both species, unusual polymorphisms in a major histocompatibility complex (MHC) class II molecule confer the most disease risk, disease is caused by perturbations by the same genes or different genes in the same biological pathways and that diabetes onset is preceded by the presence of circulating autoreactive T cells and autoantibodies that recognize many of the same islet antigens. However, the relevance of the NOD model is frequently challenged due to past failures translating therapies from NOD mice to humans and because the appearance of insulitis in mice and some patients is different. Nevertheless, the NOD mouse remains a pillar of autoimmune diabetes research for its usefulness as a preclinical model and because it provides access to invasive procedures as well as tissues that are rarely procured from patients or controls. The current article is focused on approaches to improve the NOD mouse by addressing reasons why immune therapies have failed to translate from mice to humans. We also propose new strategies for mixing and editing the NOD genome to improve the model in ways that will better advance our understanding of human diabetes. As proof of concept, we report that diabetes is completely suppressed in a knock-in NOD strain with a serine to aspartic acid substitution at position 57 in the MHC class II Aβ. This supports that similar non-aspartic acid substitutions at residue 57 of variants of the human class II HLA-DQβ homolog confer diabetes risk.

## Introduction

Since becoming available to the scientific community, the NOD mouse has been used extensively and has provided significant contributions to our mechanistic understanding of autoimmunity and type 1 diabetes (T1D). Indeed, the NOD mouse has been used to understand many facets of human T1D and has been the preferred model for invasive, preclinical/translational studies. While the NOD mouse has a number of critics, this model should be viewed as an important component of a comprehensive approach to understanding T1D. The NOD remains a standout model because it develops spontaneous T1D with genetic and environmental components that are relevant to the human disease. Further, as recent studies have demonstrated and as we describe below, new protocols to specifically modify single base pairs can generate loci that contain risk alleles that are orthologous to the human on the NOD background. Therefore, the NOD mouse remains a powerful and valuable implement in the investigator’s toolbox.

A major strength of the NOD model is the existence of spontaneous autoimmunity and T1D. Similar to the human condition, NOD mice develop autoantibodies (1) and exhibit increases in circulating autoreactive T cells (2, 3) prior to the onset of T1D. The β cell antigens that are targeted are also similar between these species (4). However, in the NOD mouse, the initiating antigen appears to be insulin (1), whereas in human T1D it is thought to result from several initiating antigens (5, 6). These autoimmune phenotypes are followed by the onset of hyperglycemia (7). A progressive loss of β cell function is present in both human and NOD mice suggesting similarities in β cell loss or dysfunction. While hyperglycemia in NOD females and males begins close to 12 and 15 weeks of age, respectively (8), immune infiltration into the pancreatic islets, insulitis, begins much earlier. Pathogenic T cells have been isolated from the islets of 5-week-old NOD mice (9). By 12 weeks of age, insulitis is present throughout the pancreas of NOD mice. A dissimilarity of the diabetes when comparing human and NOD mice is the appearance of insulitis. Studies from the nPOD bio-repository have been critical in defining insulitis in humans where this pathogenic lesion is less severe and less frequent than what can be observed in NOD mice (10). This may result from the fact that the autoimmunity in parental NOD mice is very aggressive and disease onset occurs over an abbreviated timeline (weeks) compared to the decidedly more attenuated onset in humans (i.e., years after the appearance of autoantibodies). Insulitis and T1D incidence in NOD mice can be reduced through genetic modification. While hundreds of variant NOD mice have been made that represent less intense forms of T1D, the idea of improving the NOD as a model for human T1D by decreasing the potency of the autoimmune response remains largely unexplored. The potential of this strategy is discussed below.

Genetics play a significant role in autoimmunity and important similarities exist when equating T1D-risk loci in human and NOD mice. The disease is polygenic in both species with over 50 loci linked to risk in human and NOD diabetes (11). However, a single locus is responsible for the majority of the risk: major histocompatibility complex (MHC) class II. Early papers were critical in establishing that NOD mice encode a T1D-risk MHC haplotype that has important resemblances to the HLA risk alleles in human. Since these publications, genetic and biochemical studies have linked risk to amino acid residue 57. The high-risk DQ2 and DQ8 alleles of human as well as the A^g7^ molecule of the NOD have small polar amino acids substituted for an aspartic acid at position 57. The importance of this amino acid substitution is discussed in detail by Bettini and Bettini in this issue of *Frontiers in Endocrinology* (Co-published in the same edition of FiE). The genetic variations that impart risk in HLA/MHC arise from single-nucleotide polymorphisms that change the amino acid sequence. Similarly, other genes such as *Ctla4* and *mt-Nd2* are linked to risk in both humans and NOD mice. A single leucine to methionine substitution in *mt-Nd2* as well as the human homolog, *mt-ND2*, provides β cells with enhanced resistance to autoimmune destruction (12, 13). While HLA/MHC and *mt-ND2*/*mt-Nd2* represent genes with protein and biochemical differences, these non-synonymous changes in T1D are more the exception than the rule. Only seven of the >50 single-nucleotide polymorphisms associated with T1D arise in coding regions (14). The polymorphism in *Ctla4* of NOD mice results in altered splicing. While the polymorphism in *CTLA4* is not in an identical location, the risk variant is similarly associated with altered splicing of CTLA4 (15–17). Therefore, genes such as *CTLA4* can be modeled in the NOD mouse to aid in understanding the role of non-coding genetic variation in pathogenesis of T1D. Recent advances in genetic editing have further promoted the use of NOD to understand how specific SNPs can affect protein function. Editing of the NOD genome has been used to swap T1D risk or resistance alleles allowing for the role of specific SNPs, such in MHC Class II (described below) or *Ptpn22* (18), in the regulation of autoimmunity to be identified.

Another concept that holds true across species is that T1D onset results from the sum of the genetic parts. In human subjects, T1D risk increases as the haplotype of an individual contains more credible T1D susceptibility SNPs (19, 20). Similarly, by subtracting risk loci from the NOD genome through backcrossing or genetic modification, T1D risk can be altered (11). As discussed in detail below, the NOD represents a powerful tool to study epistasis.

In the current review, we highlight past contributions NOD mice have made to T1D research and outline strategies to better utilize this model in future. Included is an overview of NOD mouse’s track record as a preclinical model for developing T1D therapies and a discussion about the impact NOD congenic mice have made to understanding the genetic basis of T1D. Also discussed is a strategy to develop panels of NOD congenic mice from existing congenic stocks to better mimic the spectrum of human autoimmune diabetes subtypes. Finally, we summarize existing and emerging technologies for editing the NOD genome that should greatly enhance the NOD mouse as a research tool, especially for identifying genes that contribute to T1D development.

## Preclinical Performance of the NOD Mouse

While the NOD mouse has proved useful in many preclinical research areas, significant tension has arisen over the performance of this mouse strain in bench-to-bedside efforts due to a failure to translate therapies developed in the NOD model to humans. The NOD model has been used for at least 30 years to identify agents or protocols that delay, prevent, or reverse disease. In general, investigators apply three approaches: early prevention (treatment is initiated at 3–4 weeks of age), late prevention (begin treatment at 10–12 weeks of age), or intervention after onset of T1D (reversal). Most preclinical successes in NOD have come in early prevention, where a wide array of agents or protocols can block disease. It should be noted that in most cases the impact of the drug/agent under investigation on autoimmunity (i.e., insulin autoantibodies or the presence of β cell reactive T cells) was not assessed. Further, many of these have seen little to no confirmation by independent laboratories. A recent NIH funded effort to confirm the effects of specific agents was unsuccessful at repeating the majority of the successes that were previously published (21). Late prevention represents a modality that is similar to trials established in humans where autoantibody positive individuals are identified and enrolled, such as the Diabetes Prevention Trial 1 or the recent oral insulin trial (22–24). To date, there has been a failure to translate late prevention successes in the NOD to prevention of human T1D.

At time of writing, very few therapies have resulted in T1D reversal in new-onset NOD mice and fewer still in NOD mice with established disease. Of the agents that have shown benefit, anti-CD3, and the combination of antithymocyte globulin (ATG) and granulocyte-colony stimulating factor (G-CSF) have been used in clinical trials. Preclinical studies using these modalities demonstrated an ability to reverse T1D in 39% of NOD females after onset (25). Multicenter preclinical efforts using anti-CD3 produced similar results, with less than 50% of the treated NOD mice exhibiting long-term T1D reversal (26). The rates of T1D reversal were significantly different when comparing sites, where anti-CD3 efficacy ranged from 10 to 80% among the four locations. Similarly, trials with anti-CD3 resulted in a minority of patients responding to therapy (i.e., preservation of c-peptide responses), yet none of the efforts with anti-CD3 resulted in insulin-free status for the patients (27–29). Likewise, use of ATG + G-CSF in a small multicenter clinical trial (25 total patients: 17 receiving ATG + G-CSF and 8 placebo) established that this combination did not induce T1D-remission but was effective in preventing erosion of β cell function 12 months after treatment (30). The 2-year data for ATG + G-CSF were less promising. At 24 months only 50% of the individuals who received therapy had preservation of β cell function (31). This is similar to the ATG + G-CSF reversal rates in NOD mice (32).

These data provide caution for moving agents forward for clinical trials that have been developed using NOD mice. A recent paper in Science Translational Medicine (33) has called for standards in clinical diagnosis as well as timing of therapy initiation in NOD mice. In preclinical studies, it is well established that NOD mice should be treated immediately after onset of T1D for maximal therapeutic response. Most groups have now established protocols for checking mice every other day for T1D onset allowing for initiation of therapy as soon as 1 day after diagnosis (21, 27, 28). In humans, trials enroll participants much more slowly and this delay in therapeutic administration likely postpones protection of the β cell mass allowing for further β cell loss. Additionally, it is clear that prior to agents or protocols moving to clinical trial there must be independent replication. The lack of a systematic understanding of T1D in the NOD and humans also impacts success. Comprehensive studies in comparative immunology and endocrinology are needed to mechanistically detail T1D reversal in NOD mice.

## Role of NOD Congenic Mice in T1D Genetics

Since researchers first started mapping *insulin-dependent diabetes* (*Idd)* loci by outcrossing NOD mice to mouse strains that do not develop T1D [i.e., C57BL/6 (B6), C57BL/10 (B10), NOR, and C3H], considerable effort has been spent creating recombinant congenic mouse strains to delineate genetic intervals containing diabetes loci and identifying the genes within each interval that are responsible for T1D susceptibility or protection. Several regions have been refined through the generation of subcongenic stocks that encode different subregions of the original confidence interval. These strains have revealed how several of the original *Idd* regions are composed of multiple susceptibility and/or resistance alleles. Notable examples include *Idd3* that was dissected into *Idd3, Idd10, Idd17*, and *Idd18* (34–37), *Idd5* that was dissected into *Idd5.1, Idd5.2, Idd5.3*, and *Idd5.4* (38–41), and *Idd9* that was dissected into *Idd9.1, Idd9.2, Idd9.3, Idd9.4*, and *Idd9.5* (42–46). While many of the dominant *Idd* regions are now well delineated, relatively few of their underlying genes have been firmly established. This is because validation has been technically challenging, in large part because even small *Idd* intervals often contain large numbers of candidate genes. Slow progress in improving candidate gene identification has led to reduced support for large-scale mouse genetic studies, forcing many in the field to decommission their congenic stocks. A new generation of genetic tools discussed in Section “Strategies for Improving Candidate Gene Identification” may reverse the fortunes of some of these strains. Nevertheless, even without discovering the causative genes, congenic mice have provided valuable insight about the genetic causes of human T1D that no other resource could have delivered. Some of their most important contributions are described below.

### Epistasis and Gene–Gene Interactions

Intercrossing congenic stocks has revealed that an individual’s disease risk is ultimately determined by the interactive effect of multiple *Idd* resistance and susceptibility loci. The challenge of disentangling these complex networks was taken up by a few courageous groups who, over decades, have detailed how different combinations of disease resistance and susceptibility loci modulate diabetes and various disease sub-phenotypes on the autoimmune-permissive NOD background. The advantage of this approach is that eliminating genetic variability between *Idd* loci allows for the detection of gene-masking and gene–gene interaction effects that are normally concealed in conventional genetic association studies with human subjects as well as mouse studies involving F2 and backcross one generation for segregation analysis (47).

There are several examples of how interactions between individual *Idd* susceptibility and resistance alleles gives rise to graded levels of diabetes on the NOD background (48–51). Among the best characterized is the interplay between the *Idd3* and *Idd5* congenic intervals from C57 strains when introgressed into the NOD genome. Combining *Idd3* and *Idd5* confers almost complete protection from T1D and insulitis on the NOD background (38). Yet, combining *Idd3* with individual *Idd5* subloci results in a spectrum of diabetes protective effects [reviewed elsewhere (11, 47, 52)]. At one end of the spectrum, *Idd3/Idd5.1* NOD mice were found not more protected against T1D than *Idd3* mice (41). Hunter et al. posited that the lack of protection in NOD-*Idd3/Idd5.1* mice may result from T1D resistance alleles at *Idd3* increasing the expression of CTLA-4 on the surface of CD4^+^ and CD8^+^ T cells that may render higher levels of inhibitory ligand-independent CTLA-4 induced by protective alleles at *Idd5.1/Ctla-4* somewhat redundant (41, 53). On the other end of the spectrum, *Idd3/Idd5.1/Idd5.3* and *Idd3/Idd5.3* recombinant congenic strains were found to exhibit T1D resistance equal to NOD-*Idd3/Idd5* mice. The lack of T1D initiation in the presence of severe insulitis observed in the *Idd3/Idd5.1/Idd5.3* and *Idd3/Idd5.3* strains indicates that the interaction between *Idd5.2/Nramp1* and *Idd3* is not important for T1D protection, but does contribute to the marked reduction in insulitis (41, 54). Continued studies of these strains will provide models to address the knowledge gap in additive and synergistic genetic effects.

Gene–gene interactions also exist among the various *Idd5* subregions, including between *Idd5.1* and *Idd5.4*. *Idd5.4* encodes a B10-derived susceptibility allele without a known responsible gene product. *Idd5.4* significantly accelerates T1D in the presence of *Idd5.2* and *Idd5.3*, but has no impact on disease if *Idd5.1* is also present (41). This suggests that *Idd5.4* can neutralize the protective effects of *Idd5.2* and *Idd5.3* and that *Idd5.4* is in turn masked by the protective effects of *Idd5.1*. A plausible explanation for this phenomenon is that immune events regulated by the B10-derived susceptibility allele at *Idd5.4* are counteracted by *Idd5.1/CTLA-4* signaling in one or more cell types. Similar masking effects have been detected among other congenic regions including between *Idd19* and *Idd6* on Chr.6 (49), *Idd19* and *Idd20* on Chr.6 (51), *Idd21.2* and *Idd21.1* on Chr.18 (50), and *Idd14* and *Idd31* on Chr.13 (55).

Evidence for epistatic interactions in humans include a study by Winkler et al. that genotyped 12 non-HLA susceptibility genes (*ERBB3, PTPN2, IFIH1, PTPN22, CLEC16A, CD25, CTLA4, SH2B3, IL2, IL18RAP, IL10*, and *COBL*) in high-risk HLA positive children of parents with T1D that were prospectively followed from birth to the development of autoantibodies and disease (19). An analysis was performed to determine the combinations of genes that most accurately predicted T1D development. The results showed that T1D progression in high-risk HLA carriers was best predicted by a collection of 8 genes (*ERBB3, IFIH1, PTPN22, CLEC16A, CTLA4, SH2B3, IL18RAP*, and *COBL*) rather than all 12 SNPs. These results suggest the presence of gene–gene interactions that mask the effect of individual diabetes susceptibility alleles. Another study searched for interactions between 38 T1D-associated non-HLA loci and different HLA class II genotypes in a large collection of T1D samples (20). It was found that SNPs within two T1D-associated genes, *PTPN22* and *CTLA4*, alter the predicted diabetes risk of various HLA haplotypes, partly confirming earlier reports that the effect of a susceptibility allele at *PTPN22* is greater in individuals expressing low-risk than high-risk HLA class II genotypes (56–58). These and other GWAS studies show how some T1D genes but not others are strongly influenced by gene-gene interactions and masking effects.

### Cellular Expression of Diabetes-Associated Genes

Congenic mice offer a powerful tool to determine how different T1D genes modulate diabetogenic immune responses within specific cell types, which cannot easily be accomplished by experimentation with human samples. Previous studies have used a variety of adoptive transfer or bone marrow chimerism methods to observe that T1D genetics regulate immune dysfunction. A good illustration is the use of the B6, B10, or NOR derived *Idd9/Idd11* resistance locus to inhibit diabetes. One set of studies found that complex genetic interactions within *Idd9/11* regulate how B cells contribute to disease by engrafting syngeneic bone marrow and B cells purified from different Chr. 4 subcongenic donors into lethally irradiated B cell-deficient and diabetes-resistant NOD.*IgH^null^* mice (59, 60). Diabetes development was then monitored to determine if B cells expressing separate subcongenic intervals from the NOR strain protected recipient mice from T1D compared to standard NOD B cells. The results established that at least four adjacent intervals interactively contribute to how diabetogenic B cells become tolerized or cause T1D, including processes that increase the efficiency of B cell anergy or B cell hyperresponsiveness to B cell receptor stimulation.

We used a similar strategy to show that genes within the *Idd9/11* locus control pathogenic CD4 T cells responses in T1D (61). Lethally irradiated CD4-deficient NOD.*CD4^null^* mice were reconstituted with syngeneic bone marrow and CD4^+^ T cells isolated from NOD.NOR-(*D4Mit31-D4Mit310*)/DvsJ: (NOD-*Idd9/11^NOR^*) NOD mice congenic for NOR genome on Chr. 4. In this system, transfer of CD4^+^ T cells isolated from NOD-*Idd9/11^NOR^* mice caused less diabetes than CD4^+^ T cells isolated from NOD. It was also shown that CD4^+^ T cells from BDC2.5 TCR transgenic mice have a reduced capacity to transfer T1D to immunodeficient NOD.CB17-*Prkdc^scid^* (NOD-*Scid*) mice when they express protective alleles at *Idd9* (62). Hamilton-Williams et al. found that CD4^+^ T cells that express protective B10 alleles at *Idd9.2* and *Idd9.3* suppress the expansion of diabetogenic CD8^+^ T cells (63). Their approach involved reconstituting NOD-*Scid* mice with purified CD4^+^ T cells from NOD or NOD.*Idd9* congenic mice co-transferred with CD4-depleted spleen and lymph node cells from NOD donors. After reconstitution, mice were infected with a vaccinia virus encoding the H-2K^d^-restricted IGRP_206–214_ epitope to measure the expansion of CD8 T cells specific for the islet antigen IGRP. High and low frequencies of IGRP-specific CD8 T cells were detected in mice, respectively, reconstituted with NOD and NOD.*Idd9* CD4 T cells indicating that *Idd9* protective alleles restore tolerance to islet IGRP through CD4 T cells.

Other cell types besides B cells and conventional CD4^+^ T cells have been found to regulate diabetes through *Idd9*. Regulatory T cells (Tregs) expressing B10-derived *Idd9.1* genes have significantly higher suppressive activity than Tregs from standard NOD mice (64). The *Idd9.1* sub-locus has also been reported to increase the capacity for DCs to engage and potentiate natural killer T cells, which are required for *Idd9*-mediated diabetes protection (65). Reciprocal transfers of NOD and NOD.*Idd9* congenic mouse spleen and lymph node cells into NOD-*Scid* and NOD.*Idd9*-*Scid* recipients identified that non-lymphoid cells possess some component of *Idd9* protection (63). Another finding was that transplanted islets from NOD-*Idd9* mice are more resistant to destruction by CD8^+^ T cells, suggesting that an element of *Idd9*-mediated T1D protection maps to insulin-producing β cells (66).

Studies dissecting the effects of *Idd9* and other T1D loci have demonstrated that diabetogenic immune responses develop from a complex interplay of genes in multiple cell types. Further, evidence suggests that different cell types can be affected by a single diabetes locus/gene with sometimes opposing effects on disease. Determining how individual *Idd* loci contribute to T1D by affecting immunoregulatory pathways in specific cells offers a useful strategy for identifying the genes underlying these regions.

### Genetic Control of Insulitis

Congenic mice have revealed that non-MHC *Idd* loci can be separated into two classes; one that supports T1D by modulating the virulence of insulitis and/or the intrinsic resistance of β cells to cytotoxic stress, and a second class that supports T1D by regulating diabetogenic immune responses before insulitis occurs (67). In the first class, replacement of individual NOD susceptibility loci with resistance alleles from non-diabetes prone strains reduces the incidence of T1D but has no quantifiable effect on insulitis at the gross histological level compared to NOD mice of the same age. *Idd* loci that fall into this category include *Idd9* where introgression of B10-derived resistance alleles did not alter the cellular composition of insulitis. Instead this locus changed the pathogenic properties of leukocytes that accumulated in islets and shifted cytokine production from IFNγ and TNFα to an IL-4 response (43). The overlapped B6-derived *Idd11* interval also reduces the pathogenic effects of β cell-specific lymphocytes in islet infiltrates without affecting the overall amount of insulitis (44). Another example is *Idd6* where C3H-derived resistance alleles confer protection against T1D but not islet infiltration. However, subtle differences exist in the invading leukocyte populations including that CD4^+^ T cells and B cells are slightly reduced, which is counterbalanced by an increase in non-lymphoid cells such as macrophages and dendritic cells (68).

Disease protection is highly variable among the second class of non-MHC *Idd* loci where resistance alleles protect against both T1D and insulitis. Some regions including *Idd10/18, Idd16*, and *Idd21* cause a mild reduction in pancreatic infiltration but only during the early phases of insulitis (34, 50, 69, 70). Most of these loci confer relatively modest protection against T1D. In contrast, loci such as *Idd3* and *Idd5* that each provide substantial diabetes protection also cause a considerable delay in insulitis, although almost all NOD.*Idd3* and NOD.*Idd5* congenic mice eventually develop significant islet infiltration (34, 38). Other *Idd* loci, including *Idd4* and *Idd13*, appear to change the distribution rather than the amount of insulitis (71, 72). NOD mice expressing either of these loci develop non-destructive peri-ductal infiltrates where invading cells remain mostly confined to the peri-islet zone until well after the age most NOD mice develop diabetes. As discussed above, none of the non-MHC *Idd* loci that block insulitis and T1D are sufficient on their own to substantially reduce islet inflammation. However, almost complete protection can be achieved when individual regions are combined, indicating that genetic interactions exist between specific loci that confer greater protection against islet inflammation than the collective effects of each separate region.

Together, these findings suggest that insulitis among patients is also under complex genetic control and that, in some people, combinations of T1D genes could cause high levels of non-destructive islet inflammation long before the onset of overt disease. In contrast, the degree of insulitis may correlate closely with progression to overt diabetes in patients that carry T1D genes that give rise to more virulent forms of insulitis.

## Modeling the Genetic Diversity of Human T1D

A major criticism of the NOD mouse has been that this model represents the equivalent of a single human case of T1D. Consequently, immune modulation protocols developed in the NOD mouse could be limited to a few subtypes of the human disease, which may partially explain why some interventions that have shown promise in NOD mice fail to preserve β cell function in patients (73). Better predictions from mouse models might be possible if future treatment protocols were screened using multicenter efforts with heterogeneous populations of NOD-derived mice to mimic the genetic variation among patients. Such a strategy could employ a panel of NOD-related recombinant congenic strains carrying different combinations *Idd* loci where each strain would express a unique set of genetic variants that give rise to a specific subtype of T1D (41). This is analogous to the different subtypes of T1D that arise in patients from various segregating combinations of susceptibility and resistance alleles. The potential of this strategy is that therapies capable of inhibiting diabetes across a panel of congenic strains are more likely to be successful in genetically heterogeneous humans. There are also advantages to finding treatments that only work in congenic mice with specific combinations of *Idd* loci, including that this could provide valuable information about the cellular and molecular mechanisms through which an immune modulation treatment affects disease. It may also help to identify specific subsets of patients that have less or more potential for responding to a particular immune therapy.

Choosing which congenic mice to include in a future drug testing panel presents a challenge because of the large number of *Idd* loci it is possible to combine. It is logical that strain selection should consider the nature of the immune modulation protocol being tested. For therapies like probiotic treatment and immune suppression protocols, where the mode of action is poorly understood or where multiple cell types and molecular pathways are involved, it may be best to test mice with a diverse array of congenic intervals designed to emulate the genetic variation in human T1D. Some of the NOD-related congenic stocks described in the Section “Epistasis and Gene–Gene Interaction” may be suitable candidates, especially those that develop NOD-like levels of T1D due to introgression of susceptibility loci from non-NOD mouse strains. A more targeted panel could be employed for therapies known to act through particular cellular or molecular pathways. For instance, immune modulation protocols designed to enhance Tregs, such as low-dose IL-2 and combined ATG + G-CSF therapy, could be tested on congenic mice expressing different allelic variants of *Idd3, Idd6, Idd9.1*, and *Idd9.3* that each separately affect the suppressive properties of Tregs (64, 68, 74, 75). Another example is antigen-specific immunotherapy where autoantigens could be screened in NOD congenic mice expressing different variants of *Il2/Il21* (*Idd3*) (74, 76), *B2m* (*Idd13*) (72), and *Ptpn22* (*Idd18.2*) (77) that, respectively, modulate T cell activation/effector function, peptide presentation, and TCR signaling. All of these factors contribute to the fate of self-reactive T cells that encounter autoantigen and may affect the outcome of autoantigen immunotherapy.

An obvious drawback to testing diabetes therapies using congenic mouse panels is the additional time and resources involved. Even so, the investment is worthwhile if therapies that are ineffectual in humans could be recognized before progressing to clinical trials. An example of how testing the appropriate NOD congenic strain might have produced a different result to standard NOD mice and predicted the failure of a T1D treatment is low-dose IL-2 therapy, which increases the frequency of Tregs but has not been able to produce positive effects on diabetes in patients (78). A chief reason that this treatment advanced to clinical trials is that low levels of IL-2 potently suppresses T1D development and reverses recent onset T1D in NOD mice, presumably by enhancing Treg function and/or development (79). However, it is possible that NOD mice are particularly sensitive to this type of immune modulation because this strain carries a variant of *Il2* that reduces IL-2 gene expression and Treg function (74). An interesting question is whether the outcome of IL-2 treatment would be different in NOD.*Idd3* mice that express the B6 variant of *Il2* and results in higher levels of *Il2* gene expression (74). The answer might address whether low-dose IL-2 therapy has potential for improving immune regulation and result in enhanced β cell function in patients without an IL2/IL2R signaling deficiency. This is important because it is still unclear whether defects in the IL2/IL2R pathway play a significant role in most cases of human diabetes; although a gene variant of *IL2RA* (CD25) has been associated with T1D risk in people, it is protective but rare (80). Furthermore, the causative gene has yet to be identified for the chromosome 4q27 region containing *IL2* and *IL21* that is linked with T1D susceptibility (81).

Another limitation of testing T1D therapies with NOD congenic mice is that many *Idd* loci strongly suppress diabetes, which will require that some experiments be performed with large numbers of animals to achieve sufficient power. Indeed, only 10–20% of female NOD.*Idd3* mice develop T1D by 30 weeks of age (74, 76, 82). As mentioned above, the unique insights from congenic mice will often justify using strains with very low levels of disease. However, there is also potential to alter the genetic composition of congenic strains in ways that will increase the rate of diabetes. For instance, it may be feasible to use NOD mice heterozygous instead of homozygous for the *Idd3* locus, which develop 40% diabetes (76). These mice still produce more IL-2 than standard NOD mice and would presumably be less sensitive to IL-2 therapy. Another strategy could be to breed T1D-resistant congenic strains with NOD mice carrying congenic intervals that accelerate diabetes. For example, NOD.*Idd3* could be crossed to NOD mice carrying B6 alleles at *Idd18.2*/*Ptpn22* that are more diabetogenic than the corresponding NOD alleles (77).

## Strategies for Improving Candidate Gene Identification

Although genetic studies using inbred mice are costly because of the large number of mice required, they remain a powerful method of detecting rare T1D susceptibility alleles that are impractical to identify through GWAS analyses, which require tens or hundreds of thousands of human subjects (83, 84). Thus, for the reasons outlined above, the question is not whether pursuing the identity of T1D susceptibility and resistance alleles is worthwhile, but rather how to make this process more efficient by employing a comprehensive approach that utilized both human and mouse systems. Considerable encouragement comes from a new generation of genetic tools that may circumvent many of the most intractable obstacles that traditionally limited the identification of *Idd* candidate genes. Some of these are described in the following sections in the order of their development.

### RNA Interference (RNAi)

RNA interference has proven useful for manipulating gene expression in NOD mice without introducing genetic contamination from other strains. This approach is based on a well-established transgenesis methodology that entails the direct introduction of short hairpin RNA (shRNA) containing constructs into NOD zygotes by viral transduction (85, 86). The shRNA-containing constructs are designed to silence genes that impact T1D. shRNA is a sequence of RNA that contains a tight hairpin turn. This structure is cleaved by intracellular machinery into small interfering RNA that knocks down any mRNA bearing a complementary sequence (87). Several companies are developing viral libraries that produce shRNA that integrate into the host genome and ensure stable gene silencing after integration. The silencing cassette can be incorporated into many different types of vectors, including lentiviral, adenoviral, or retroviral vectors. Using the NOD model, RNAi has already provided valuable insight into how expression of the T1D candidate genes *IL17* (88), *PTPN22* (89), *CTLA4* (90), *CLEC16A* (91), *RGS1* (92), and *Slc11a1* (*Nramp1*) (93) contribute to diabetes development. It is conceivable that T1D susceptibility genes can regulate disease progression in an age-dependent manner. Establishment of inducible RNAi has also enabled temporal control of target gene knockdown to determine their functions at different stages of disease progression (94).

### Zinc Finger Nuclease (ZFN)

Zinc finger nucleases are fusion proteins containing a sequence-specific DNA-binding zinc finger domain and a nuclease domain (95, 96). Engineered ZFNs specifically recognize and bind a defined target gene sequence within the nucleus of a cell and introduce a double-strand break (DSB) (97, 98). The cellular DNA repair machinery fixes these breaks, most frequently *via* the non-homologous end joining (NHEJ) mechanism resulting in small deletions or insertions of the gene sequence (few to hundreds of base pairs) and disruption (knockout) of the target gene (97, 98). Injected as synthetic mRNAs, ZFNs typically work at the one-cell fertilized embryo stage, resulting in single-step, whole animal gene disruption, and infrequent mosaics (99). More precise genetic engineering can be achieved as well because a DSB also stimulates DNA repair *via* homology-directed repair (HDR) mechanism if a homologous DNA template is co-introduced into the cell (100). Because ZFN-mediated genetic manipulation can be done directly in NOD embryos, the resulting knock-in or knockout can be generated on a pure NOD genetic background. Thus, it eliminates carryover of closely linked passenger DNA that occurs when the induced mutation is introduced in non-NOD embryonic stem cells (129 or B6) and then the targeted allele is backcrossed onto NOD. This is particularly concerning when targeting genes within known *Idd* regions. To study the role of the *Idd9.3* candidate gene *Tnfrsf9* (encoding CD137/4-1BB), we used ZFN to disrupt this gene directly in NOD embryos (101). The NOD allele of CD137 is hypofunctional when compared to the B10 protein that is expressed within the *Idd9.3* congenic strain (102). Thus, it was thought that T1D development would be accelerated in the absence of CD137. Surprisingly, CD137-deficient NOD mice were less susceptible to T1D, indicating that this co-stimulatory molecule has a diabetogenic role. This conclusion could not have been made with certainty if CD137-deficient NOD mice were created by backcrossing the previously reported knockout alleles generated using 129 embryonic stem cells. We further established an important role of CD137 in promoting the accumulation of β cell autoreactive CD8^+^ T cells (103). In addition, CD137 had a diabetes protective function when expressed in CD4^+^ T cells, likely due to the immunosuppressive activity of soluble CD137 produced by Tregs (103).

**Figure 1:**
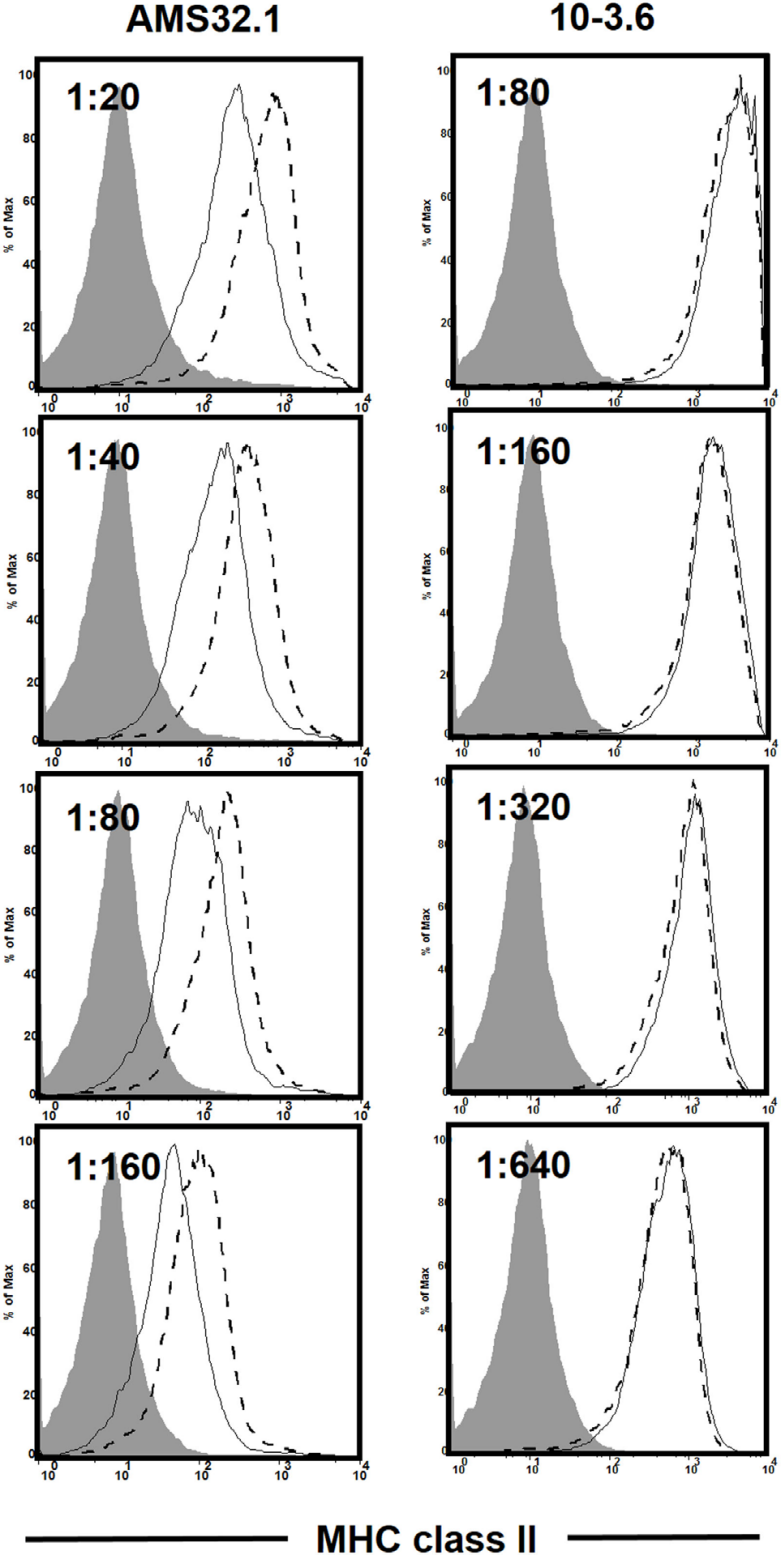
Differential staining of the major histocompatibility complex (MHC) class II molecule in wild-type NOD and NOD.*Ab^g7-S57D^* mice. Total splenocytes were stained with anti-CD11c, anti-CD19, and the indicated I-Ab antibody clone at different titers. Shown is the I-Ab staining on B cells (CD19^+^ CD11c^−^) of NOD (dashed line) and NOD.*Ab^g7-S57D^* (solid line) mice. The shaded area is the negative control staining using splenocytes isolated from I-Ab deficient NOD mice (NOD/ShiLtJ-*H2-Ab1^em1Ygch^*/J, JAX stock no. 027057). Similar results were obtained in two independent experiments.

**Figure 2:**
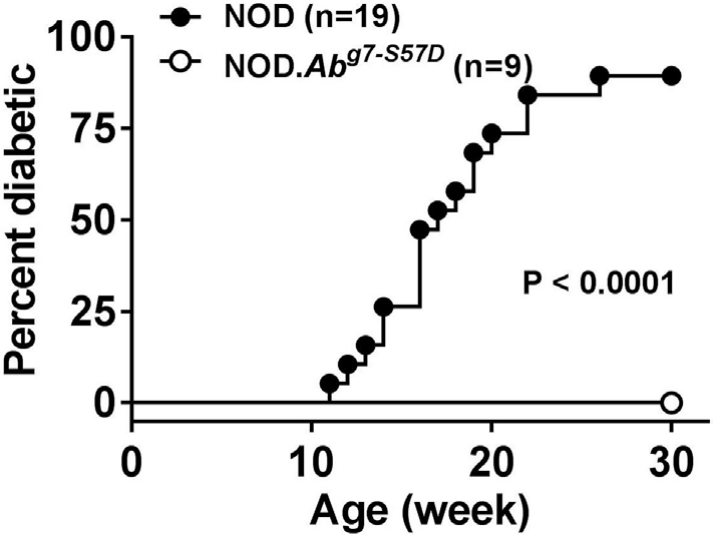
NOD.*Ab^g7-S57D^* mice are completely resistant to type 1 diabetes. NOD and NOD.*Ab^g7-S57D^* mice were monitored for diabetes development weekly for 30 weeks by testing urine glucose. Diabetes onset was defined by two consecutive readings of >250 mg/dl.

As discussed earlier, the *H2^g7^* haplotype is essential for T1D development in NOD mice. A key component of the diabetogenic *H2^g7^* haplotype is the unique *Ab^g7^* allele. The *Ab^g7^* allele includes five nucleotide polymorphisms resulting in the conversion of two usually conserved proline and aspartic acid residues at positions 56 and 57 to histidine and serine (104). Significantly, the non-aspartic acid substitutions at residue 57 also characterize diabetogenic variants of the human class II HLA-DQβ homolog, such as DQ8 (105). While transgenic analyses have shown both histidine and serine, respectively, at positions 56 and 57 amino acid residues of Aβ^g7^ to be important for T1D progression in NOD mice (106–108), their diabetogenic function has not been tested under a more physiological condition. To further study the role of amino acid residue at position 57 in Aβ^g7^, we created a knock-in NOD strain by replacing the serine with an aspartic acid (Aβ^g7^-S57D). This was achieved by co-injecting *Ab^g7^*-specific ZFN coding mRNA and a plasmid construct for HDR into one-cell fertilized NOD embryos, which were subsequently transferred into pseudopregnant mothers, and live-born pups were screened for founders. We successfully established a knock-in NOD stock (formal name: NOD/ShiLtJ-*H2-Ab1^em2Ygch^*/Ygch) with the precise 3 base pair alteration resulting an aspartic acid at position 57 in the MHC class II Aβ chain. The knock-in allele was confirmed at both the genomic DNA and cDNA levels. We used two different antibody clones (AMS-32.1 and 10-3.6) to determine if MHC class II expression was altered in NOD.*Ab^g7-S57D^* mice. The expression level of MHC class II on B cells and dendritic cells was found to be comparable in wild-type NOD and NOD.*Ab^g7-S57D^* mice when 10-3.6 was used to stain their splenocytes (Figure 1 and not shown). Interestingly, the level of MHC class II staining was found to be lower on B cells and dendritic cells from NOD.*Ab^g7-S57D^* than those from wild-type NOD mice when AMS-32.1 was used (Figure 1 and not shown). These results indicate that the aspartic acid substitution at position 57 in the Aβ chain alters the binding of AMS-32.1, presumably due to a conformational change of the antibody-binding epitope. Strikingly, diabetes development was completely suppressed in homozygous NOD.*Ab^g7-S57D^* mice (Figure 2), confirming the importance of the aspartic acid residue at position 57 of the Aβ chain in T1D. The availability of this novel strain will allow studies aimed to understand how diabetogenic MHC class II molecules select and activate β-cell autoreactive CD4 T cells.

### Clustered Regularly Interspaced Short Palindromic Repeat (CRISPR) and CRISPR-Associated Protein 9 (Cas9)

Due to its high efficiency, the CRISPR/Cas9 system has become the top choice when considering gene targeting in a variety of animal models. Similar to ZFN mediated mutagenesis, CRISPR/Cas9 also introduces a DSB, followed by repair through NHEJ and HDR dependent mechanisms (109). Cas9 nuclease is recruited to a specific DNA sequence by a single-guide RNA that can be easily designed using publically available online tools (109). Several groups, including ours, have successfully used the CRISPR/Cas9 approach to disrupt genes directly on a pure NOD genetic background (18, 110–112). The importance of affinity maturation processes of B cells (class switch recombination and somatic mutation) for T1D development in NOD mice was demonstrated by ablation of the activation-induced cytidine deaminase gene (*Aicda*) (111). It was recently shown that IL-2 can indirectly enhance FOXP3 expression through downregulating the level of *Flicr*, a long non-coding RNA (112). The function of Tregs is impaired in NOD mice partly due to reduced IL-2 production by activated T cells in this strain (74). Deletion of *Flicr* decreased accumulation of FOXP3^low^ Tregs in pancreatic islets and suppressed T1D in NOD mice, likely by enhancing the stability and function of Tregs (112). *Ptpn22* has also been targeted in NOD mice using the CRISPR/Cas9 system (18). A nonsynonymous single nucleotide polymorphism resulting in an amino acid substitution (R620W) in human PTPN22 has been linked to numerous autoimmune diseases, including T1D (113). *Ptpn22* has been identified as a top candidate gene for the *Idd18.2* region in NOD mice. To further study the role of PTPN22 in T1D, the Sherman laboratory generated a *Ptpn22* knockout NOD mice as well as a knockin strain that has the R619W amino substitution to mimic the human variant (18). *Ptpn22* knockout NOD females developed more rapid onset of T1D (18). Similarly, NOD females expressing the *Ptpn22* KI allele (encoding 619W) also developed accelerated T1D (18), providing direct evidence to support the diabetogenic function of this variant.

### Testing Human T1D Candidate Genes in NOD Mice

As noted above, the NOD mouse has been criticized for its usefulness as an animal model for human T1D, largely due to disappointing outcomes of clinical trials based on agents that showed therapeutic and/or preventive effects for mouse diabetes. The increased availability of human samples allowing direct examination of pancreata and lymphoid tissues isolated from organ donors at different stages of T1D progression has further decreased the enthusiasm of the NOD model (114). However, it remains a challenge to identify and to mechanistically study T1D susceptibility genes in human. The effect of a single gene on a phenotype is more difficult to detect due to heterogeneity in humans. Many genetic variations associated with T1D have a low phenotypic impact that overlaps when comparing carriers and non-carriers. In addition, human studies are mostly association in nature and strategies that allow investigators to directly analyze the diabetogenic function of a single SNP alone or in combination are limited. The CRISPR/Cas9 system makes it possible to engineer isogenic cell systems that can be used to specifically address the role of a SNP in gene expression and function (115). When combined with the ability to generate patient-derived iPSC and the advance of *in vitro* differentiation of iPSC into insulin-producing β-cells and hematopoietic stem cells, it may be possible to test the function of a SNP in cell types relevant to T1D (116, 117). However, these studies are not likely to overcome the difficulty to understand the course from altered gene expression/function to T1D development, which can only be dissected with *in vivo* experimental systems as disease progression is a consequence of combined effects that a variant elicits in different cell types in a time-dependent fashion. Combinational approaches using both mouse and human experimental systems are thus required to have a comprehensive understanding of the genetic control in T1D. The ability of nuclease based technology to efficiently and precisely modify the genome directly in NOD mice has opened a new door for current and future T1D genetic studies using this model.

Because T1D is a complex disease influenced by a large number of genes and ill-defined environmental factors, the NOD mouse remains an ideal animal model that provides a disease susceptible genetic background to test the diabetogenic function of a human candidate gene. For this reason, we have used both ZFNs and CRISPR/Cas9 systems to target mouse orthologs of human T1D candidate genes nominated by GWAS. As discussed above, the availability of the nuclease based technologies made it possible to do a relatively small scale but focused screening for genes that can regulate T1D progression in NOD mice. We have successfully targeted more than 40 genes directly in NOD mice. While these studies are still ongoing, the results obtained from this screening will allow us to provide additional evidence to support their roles in human T1D and prioritize them for future mechanistic studies. The eventual goal is to identify a pathway that could be pharmaceutically targeted for clinical translation.

## Conclusion Remarks

Despite some shortcomings, NOD mice and NOD-derived recombinant congenic strains provide many advantages for T1D research. As discussed above, the NOD mouse continues to be an important tool for dissecting the genetic control of T1D. As will be discussed below, we also describe T1D research areas where NOD mice and related strains can provide critical information in the next decade.

Previous studies have generated NOD mice transgenically expressing HLA class I and II molecules associated with human T1D (118–121). While HLA class II molecules in NOD mice are not able to promote T1D, expression of the HLA A2.1 allele accelerates diabetes development, providing a model for identifying peptides targeted by A2.1-restricted CD8^+^ T cells and for testing antigen-specific immunotherapy (122, 123). When combined with various versions of the severe immunodeficient NOD mice (e.g., NSG mice), expression of HLA class I or II molecules in the absence of murine counterparts provides a superior host for primary human T cells and hematopoietic stem cell-derived immune system (124). NSG mice that also express high-risk HLA class I or II molecules have been transplanted with human peripheral blood mononuclear cells or β-cell autoreactive T-cell clones/lines isolated from T1D patients to test the diabetogenic potential of the presumably pathogenic effectors (125–127). Although much progress has been made, overt diabetes has not been observed in HLA class I or II expressing NSG mice transfused with human T cells in various settings. The eventual goal is to reconstitute a T1D prone human immune system that targets β cells derived from the same subject in a mouse for studying “human” T1D. Recent advance in differentiating human iPS cells into functional insulin-producing β-cells and hematopoietic stem cells has brought us one step closer to this goal (116, 117).

Gut microbiome has emerged as an important component that modulates the progression of T1D in both humans and NOD mice (128–134). Longitudinal studies in humans showed that alteration of the diversity and species of gut microbiota preceded T1D onset (133). Studies in NOD mice have shown that manipulation of gut microbiota by means of antibiotics, fecal transfer, or co-housing can either suppress or promote diabetes development (128, 135–138). Collectively, these studies demonstrate that the NOD mouse can provide an excellent experimental platform for understanding the roles of gut microbiota in T1D. Recent studies also suggest that T1D modulation by gut microbiota is not likely to be caused by a single species but rather due to the balance of diverse species within the bacterial community. While it remains to be tested, experiments that utilize germ-free NOD mice reconstituted with fecal samples from T1D patients, at risk individuals, and healthy subjects may provide some information regarding the “good” and “bad” gut bacterial community. This knowledge can then be used to develop methods to alter the gut microbiota for T1D prevention and set the foundation for future clinical trials.

## Ethics Statement

All animal studies were approved by the Institutional Animal Care and Use Committee at the Medical College of Wisconsin under protocol AUA00001863.

## Author Contributions

All authors listed have made a substantial, direct, and intellectual contribution to the work and approved it for publication.

## Conflict of Interest Statement

The authors declare that the manuscript was written in the absence of any commercial or financial relationships that could be construed as a potential conflict of interest.
